# Theranostic profiling for actionable aberrations in advanced high risk osteosarcoma with aggressive biology reveals high molecular diversity: the human fingerprint hypothesis

**DOI:** 10.18632/oncoscience.21

**Published:** 2014-03-12

**Authors:** Daniela Egas-Bejar, Pete M. Anderson, Rishi Agarwal, Fernando Corrales-Medina, Eswaran Devarajan, Winston W. Huh, Robert E Brown, Vivek Subbiah

**Affiliations:** ^1^ Division of Pediatrics, The University of Texas MD Anderson Cancer Center, Houston, TX; ^2^ Pediatric Hematology/ Oncology/ BMT, Levine Children’s Hospital/ Levine Cancer Institute, Charlotte, NC; ^3^ Department of Investigational Cancer Therapeutics (Phase I Clinical Trials Program), Division of Cancer Medicine, The University of Texas MD Anderson Cancer Center, Houston, TX; ^4^ Department of Orthopedic Oncology- Research, The University of Texas MD Anderson Cancer Center, Houston, TX; ^5^ Department of Pathology, UT Health-Department of Pathology and Laboratory medicine, Houston, TX

**Keywords:** Osteosarcoma, Targeted therapy, Sarcoma, Bone tumors, Next generation sequencing, CLIA, biomarker

## Abstract

The survival of patients with advanced osteosarcoma is poor with limited therapeutic options. There is an urgent need for new targeted therapies based on biomarkers. Recently, theranostic molecular profiling services for cancer patients by CLIA-certified commercial companies as well as in-house profiling in academic medical centers have expanded exponentially. We evaluated molecular profiles of patients with advanced osteosarcoma whose tumor tissue had been analyzed by one of the following methods: 1. 182-gene next-generation exome sequencing (Foundation Medicine, Boston, MA), 2. Immunohistochemistry (IHC)/PCR-based panel (CARIS Target Now, Irving, Tx), 3. Comparative genome hybridization (Oncopath, San Antonio, TX). 4. Single-gene PCR assays, *PTEN* IHC (MDACC CLIA), 5. UT Houston morphoproteomics (Houston, TX). The most common actionable aberrations occur in the *PI3K/PTEN/ mTOR* pathway. No patterns in genomic alterations beyond the above are readily identifiable, and suggest both high molecular diversity in osteosarcoma and the need for more analyses to define distinct subgroups of osteosarcoma defined by genomic alterations. Based on our preliminary observations we hypothesize that the biology of aggressive and the metastatic phenotype osteosarcoma at the molecular level is similar to human fingerprints, in that no two tumors are identical. Further large scale analyses of osteosarcoma samples are warranted to test this hypothesis.

## INTRODUCTION

Osteosarcoma is the most common primary malignant bone tumor in children, adolescents, and young adults [1]. Among patients with non-metastatic osteosarcoma, the introduction of adjuvant and neoadjuvant therapy with systemic cytotoxic agents such as cisplatin, doxorubicin, ifosfamide, and methotrexate has resulted in long-term event-free survival rates of 65–75% [1-3]. Still, one-third of osteosarcoma patients develop recurrent disease[3]. These patients’ treatment options are limited to the complete surgical resection of clinically detectable disease with or without chemotherapy, and their prognosis is extremely poor[4]. The survival benefit offered by current standard cytotoxic chemotherapy regimens has plateaued, and U.S. Food and Drug Administration–approved molecularly targeted therapies for osteosarcoma are non-existent [5]. Researchers have attempted to overcome this challenge by studying the pathogenesis of patients’ tumors. However, previous molecular studies to elucidate the pathogenesis of osteosarcoma have been difficult as a result of the enormous genetic instability of the disease, which obscures the identification of common genetic loci involved in its genesis. Bench to bedside translations of molecular profiling studies from pre-clinical models of osteosarcoma are challenging owing to the polyclonal and heterogenous biology in clinical disease as opposed to a monoclonal homogenous disease in pre-clinical models.

Patients with relapsed osteosarcoma are often referred to targeted therapy clinics for enrollment in early-phase clinical trials. Therapy targeting specific molecular aberrations is relevant even in phase I clinical trials [6], and identifying targetable aberrations may be the foundation for developing potential targeted therapies, especially for “orphan” diseases such as osteosarcoma. The recent exponential growth of theranostic molecular profiling services has led to the widespread availability of Clinical Laboratory Improvement Amendments (CLIA)- certified molecular profiling. These commercially available profiling services that include next generation sequencing based assays, FISH assays, immunohistochemistry based morphogenomic (morphology plus genomics) and morphoproteomic (morphology plus proteomics) analyses are valuable tools clinicians can use to customize therapy for individual patients [7]. Previous studies have reported that even in heavily pre-treated patients, identifying actionable aberrations and matching them to molecularly matched therapies based on these abnormalities yields a better response than to unmatched therapies[8]. Targeted therapies have significantly altered the landscape of oncology, for instance BRAF inhibitors in patients with *BRAF*-mutated melanoma [9] and ALK inhibitors in patients with *ALK*-rearranged lung cancer [10]. However, few studies have used clinical molecular profiling to identify targetable aberrations in osteosarcoma. The purpose of the present study was to assess the molecular profiles of patients with relapsed advanced osteosarcoma to identify pathway aberrations that might be targeted with existing or developing therapies. The most common actionable aberrations found occur in the *PI3K/PTEN/mTOR* pathway. No patterns in genomic alterations beyond the above are readily identifiable, and suggest both high molecular diversity in osteosarcoma and the need for more analyses to delineate distinct subgroups of osteosarcoma defined by genomic alterations. Based on our preliminary observations we hypothesize that the biology of the aggressive and metastatic phenotype of osteosarcoma at the molecular level is similar to human fingerprints, in that no two tumors are identical with the caveat that this is based on a limited number of patients.

## RESULTS

Twenty patients with diagnosis of osteosarcoma were referred to Phase I Clinical Trials Department of Investigational Cancer Therapeutics (Phase I Clinical Trials Program) for evaluation between 6/1/2008 and 02/01/2013. From these, only 13 patients (7 males and 6 females) had archival tumor tissue available with molecular profiling results. The median age at initial presentation was 18 years (range, 9-46 years). Most patients (58%) had osteoblastic osteosarcoma. The most common primary tumor locations were the pelvis in 3 patients (25%), the femur in 3 patients (25%), and the tibia in 3 patients (25%). Eleven patients (92%) had metastasis to the lungs, while 6 patients (50%) had metastasis to both the lungs and bones, and 1 patient (8%) had metastasis to the bones only. The median number of prior therapies was 6 (range, 2-11 therapies). It is interesting to note that 11 out of the reported 13 patients were Caucasian. The most common aberrations identified were loss of the phosphatase and tensin homolog gene, *PTEN*, (3 patients; 25%) and mutations in class IA p110-alpha catalytic subunits of the phosphatidylinositol 3 kinase gene, *PIK3CA* (2 patients; 17%).

One patient’s tumor specimen was positive for NESTIN gene. Two patients (17%) had a mutation or loss of the retinoblastoma gene, *RB1*, and 2 patients had *TP53* truncation or mutation. Other mutations identified included *MYC* amplification, *BCL2L2* amplification, *NKX2-1* amplification, *EGFR* amplification, *JUN* amplification, and *CCNE1* amplification, as well as mutation of the protein tyrosine phosphatase delta gene, *PTPRD*, and amplification of *MET* or the hepatocyte growth factor receptor gene, *HGFR*. Five patients (patients 1, 2, 3, 4, and 9) had no identified pathway aberration. One patient with osteosarcoma of the jaw was positive for CD30 by immunohistochemistry staining.

**Figure 1:**
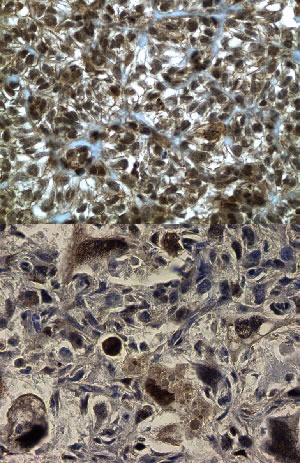
Morphoproteomic analysis of mTOR expression: First figure (patient 8) shows activation of mTOR pathway in all tumor cells as evidenced by phosphorylation of mTOR at serine 2448, at an intensity of 1-2+, occasionally nuclear, but mostly plasmalemmal and cytoplasmic. Second figure (patient 10) shows activation of the mTOR pathway in a minor component of the tumor cells with both nuclear and cytoplasmic-plasmalemmal expression of pMTOR (Ser 2448). This indicates both mTORC2 and mTORC1 activation, but again only in a minor component of the tumor

Immunohistochemistry-based morphoproteomic analyses of the tumor specimens from 2 patients (8 and 10) revealed evidence of *MAPK* pathway activation via *p-ERK1/2* and *mTOR* pathway activation via *p-mTOR*, as well as heat shock protein 90 (HSP90), tumor necrosis factor–related apoptosis-inducing ligand (TRAIL), and *nestin* expression. Ninety-eight percent of patient 8’s tumor cells expressed fatty acid synthase, and 50% of patient 10’s tumor cells expressed insulin-like growth factor. Figure 1 shows immunohistochemistry expression of *p-mTOR* in patient 8 and 10.

Table 1 summarizes characteristics of patients with advanced osteosarcoma and pathway aberrations found. Table 2 presents pathway aberrations identified in the 12 patients in a schematic fashion.

**Table 1:** Characteristics of patients with advanced osteosarcoma and pathway aberrations

Patient	Age, years	Histology	Primary tumor location	Metastases	No. of prior therapies	Profiling source	Pathway aberration
1	30	Telangiectatic	Right humerus	Lungs	5	MDA	None
2	46	Osteoblastic	Left pelvis	Lungs	8	MDA	None
3	20	Osteoblastic	Left tibia	Bones, lungs, intraabdominal, soft tissue	6	MDA	None
4	17	Chondroblastic	Pelvis	Lungs, local, bone	11	MDA	None
5	17	Osteoblastic	Right humerus	Lung, bones	11	C	*PTEN* loss BRCA1 overexp, ERCC1 overexp RRM1 overexp, TOPO1 overexp TOPO2A overexp, TS overexp
						F	*PTEN* loss *MYC* amp, *TP53* trun, *BCL2L2* amp,*NKX2-1* amp, *RB1* loss
6	12	Fibroblastic	Left tibia	Lung, local	2	F	*PTEN* loss
						C	*EGFR* mut, *PTEN* loss Sparc overexp, ERCC1 Overexp TOPO1 overexp, TOPO2 overexp HER2/neu overexp
7	17	Osteoblastic	Left tibia	Lung	5	F	*PTEN* loss *PIK3CA* mut, *TP53* mut, *RB1* mut, *JUN* amp,
8	9	Osteoblastic	Right femur	Lung, bones	7	M	*p-ERK1/2* exp, *p-mTOR* exp, *HSP 90*exp, *TRAIL* moderate exp, *NESTIN* exp
9	18	Osteoblastic	Left femur	Lung, lymph nodes, bones, suprarenal, subcutaneous tissue	6	MDA	None
10	14	Chondroblastic	Right fibula	Lung	6	M	*p-ERK1/2* exp, *p-mTOR* exp, *HSP 90* exp, *TRAIL* exp, *NESTIN* exp, *IGFR* exp
11	17	Chondroblastic	Right femur	Lung	5	F	*MET* amp, *PIK3CA* mut, *CCNE1* amp, *PTPRD* mut
12	19	Osteoblastic	Right ileum and acetabulum	Bone	9	C	TLE3 HER/2neu
						O	*SOX2* amp, *FGFR1* amp
13	19	Osteoblastic	Right iliac wing, T9 and mandible	Bone, Lung	6	Q	CD 30+

C: Caris Life Sciences; CL: Clarient, Inc.; F: Foundation Medicine; MDA: MD Anderson CLIA-compliant laboratory; M: Morphoproteomics; O: Oncopath. Exp= expression, amp= amplification, mut= mutation, overexp= overexpression, Q: Quest diagnostics

**Table 2:** Schematic representation of Pathway aberrations (mutation, enzymatic upregulation) identified in the twelve advanced osteosarcoma patients. Patient # 13 had CD 30 + per IHC (not included).

	Patient											
Aberration	1	2	3	4	5	6	7	8	9	10	11	12
PTEN loss												
BRCA1												
ERCC1												
RRM1												
TOPO1												
TOPO2A												
TS												
MYC												
TP53 mut/trun												
BCL2L2												
NKX2-1												
RB1 lpss/mut												
EGFR												
HER/2neu												
PIK3CA												
JUN amp												
p-ERK1/2												
p-mTOR												
HSP90												
TRAIL												
NESTIN												
IGFR												
MET												
CCNET1												
PTPRD												
TL3												
SOX2												
FGFR1												

Patient 1 was an Asian female diagnosed at age 30 years with osteosarcoma of the right humerus. After undergoing partial tumor resection, patient was referred to our institution, where she received 4 cycles of doxorubicin plus intra-arterial cisplatin, followed by radical resection and reconstruction of the right humerus. Patient developed lung metastases which progressed despite chemotherapy with high dose ifosfamide, doxorubicin, therapy with bevacizumab and temsirolimus, and bevacizumab, cedarinib and carboplatin and trientine, local radiation therapy and resection of metastases. Patient died at age 32 of disease progression. PCR-based DNA sequencing analysis of the patient’s tumor specimen revealed no *EGFR, BRAF, KRAS, PI3KCa,* or *c-kit* mutations.

Patient 2 was a Caucasian female diagnosed with osteosarcoma of the left pelvis at age 46 years. She was found to have a solitary lung nodule after 6 cycles of doxorubicin and cisplatin, and after resection of this nodule was disease-free for almost 3 years. Patient had a recurrence in the lungs and pelvis. She progressed despite chemotherapy with methotrexate. Moreover, she did not tolerate ifosfamide (neurotoxicity), irinotecan +erlotinib (diarrhea) and also progressed on *mTOR* inhibitor therapy. The patient had local radiation and surgery on the left hip. She progressed in the pelvis despite doxorubicin, and trial therapy with lapatinib and metformin. Patient discontinued treatment, dying at age 56 years. PCR-based DNA sequencing analysis of the patient’s tumor specimen revealed no *EGFR, BRAF, KRAS, NRAS, PI3KCa,* or *c-kit* mutations.

Patient 3 was a Hispanic male diagnosed at age 20 years with osteoblastic osteosarcoma of the left tibia with multiple bone, lung, and abdominal metastases. He had initial good response to doxorubicin, methotrexate and cisplatin but then progressed on this regimen and also on regimens containing high-dose ifosfamide, high dose methotrexate, gemcitabine with docetaxel, bevacizumab, zoledronic acid, denosumab, temsirolimus with gemcitabine, an aurora kinase inhibitor trial, followed by single agent docetaxel, sorafenib and denosumab. The patient died of disease progression at age 21 years. Molecular profiling of the patient’s tumor did not reveal pathway aberrations.

Patient 4 was a Caucasian male diagnosed with left pelvis osteosarcoma at age 17 years. He had a subcentimeter lung nodule at diagnosis. He responded minimally to doxorubicin, ifosfamide and methotrexate, and his chemotherapy was changed to doxorubicin, cisplatin, and high-dose methotrexate. He underwent hemipelvectomy and then received chemotherapy with high-dose ifosfamide. Patient developed multiple lung metastases, and after partial resection of these metastases, he was treated with gemcitabine and muramyl tripeptide phosphatidylethanolamine (MTP-PE) but developed local recurrence. Surgical resection of local recurrence was done and chemotherapy with MTP-PE with the addition of rapamycin and metformin. He also received radiation to the lungs. Patient developed spinal metastases and was treated with temozolomide and received local radiotherapy. He died of disease progression at age 20 years. No significant aberrations were found when tumor profile was performed.

Patient 5 was a Caucasian male diagnosed with osteosarcoma of the right humerus at age 17 years after he sustained a pathologic fracture. The patient also had lung metastases upon his initial presentation and was treated on a high-risk protocol that included doxorubicin, ifosfamide, cisplatin, etoposide and high-dose methotrexate. While on this regimen, he underwent limb salvage surgery and bilateral thoracotomy with resection of lung metastases. He then received therapy with liposome-encapsulated MTP-PE; while on this regimen, he underwent a second bilateral thoracotomy with resection of lung metastases. Unfortunately, this surgery revealed additional new metastases on the left lung for which left thoracotomy was performed later and after this patient was treated with gemcitabine and docetaxel. Positron emission tomography scan revealed osseous metastases to the ribs and sternum. The patient continued gemcitabine and docetaxel, the latter alternating with bevacizumab. He received radiation plus temozolomide for local disease control but developed chronic osteomyelitis, requiring a prolonged course of antibiotics. After finishing radiotherapy, the patient received sorafenib but had to discontinue it owing to significant fatigue. He was found to have *HER2/neu* positivity and received an infusion of chimeric antigen receptor T cells directed against HER2/neu. Patient had disease progression on these therapies and also on pazopanib and lapatinib, dying at 21 years of age. Patient had 2 tumor profiles performed on metastatic lung lesions. Caris analysis revealed PTEN loss by immunohistochemistry and RT-PCR analysis showed high expression of BRCA1, excision repair cross-complementation group 1 (ERCC1), ribonucleotide reductase subunit M1 *(RRM1),* Topoisomerase 1 *(TOPO1),* topoisomerase 2 alpha *(TOPO2A)* and thymidylate synthase *(TS).* Foundation next generation sequencing (NGS) based assay revealed *PTEN* loss, *MYC* amplification, *TP53* truncation, *BCL2L2* amplification, *NKX2-1* amplification, and *RB1* loss.

Patient 6 was a Caucasian male diagnosed with left distal tibia osteosarcoma with pulmonary metastases at age of 12. He received standard chemotherapy with methotrexate, cisplatin, and doxorubicin. He underwent limb salvage surgery and had 100% necrosis of the primary tumor. He completed 30 weeks of chemotherapy and then underwent thoracotomy with resection of the lung metastases, which also showed 100% necrosis. Twelve years after initial diagnosis patient was found to have biopsy-proven local recurrence. He received 4 or 5 cycles of cisplatin, high-dose methotrexate, and doxorubicin, underwent above-the-knee amputation of the left leg and then received 2 cycles of ifosfamide and etoposide. He was later found to have biopsy proven lung metastases and progressed on doxorubicin and high-dose ifosfamide, rapamycin and metformin, discontinuing therapy owing to severe side effects (joint pain, mouth sores, nausea and vomiting). The patient was referred to our Phase 1 Clinical Trial Service where his tumor was sequenced and two molecular profiles were performed. Foundation NGS sequencing of the patient’s primary tumor revealed *PTEN* loss. The immunohistochemistry profile performed by Caris revealed overexpression of Sparc polyclonal, Sparc monoclonal, *ERCC1, TOPO1, TOPO2A, PTEN,* and *HER2/neu*. The RNA microarray revealed overexpression of *VDR, ERCC1*, and *TOPO2A* and *TS*. Interestingly, fluorescence in situ hybridization revealed *EGFR* positivity in the 100 tumor cells counted. The patient had not yet received any *EGFR*-based therapies and was therefore started on cetuximab. However, the patient died of disease progression at age 28 years.

Patient 7 was a Caucasian male diagnosed with osteosarcoma involving the left proximal tibia at age 17 years. He initially received chemotherapy consisting of methotrexate, intraarterial platinum, and doxorubicin. Limb salvage surgery was performed and tumor necrosis estimated to be 95%. Adjuvant chemotherapy included carboplatin, doxorubicin, ifosfamide, and high-dose methotrexate. At the end of this therapy, imaging studies showed indeterminate lung nodules 3 of which were resected and showed a low percentage of necrosis. After 6 months of treatment with gemcitabine, he was found to have a new lung lesion which was resected. He then received 48 doses of liposome-encapsulated MTP-PE but despite this developed a peripheral left lung lesion that was resected. He eventually developed multiple lung nodules, some of which were excised. This recurrence was treated with liposomal doxorubicin, and the patient was referred to our Phase 1 Clinic Trial Service where two different tumor profiles were performed. Foundation analysis (NGS) of lung metastases specimen revealed *PIK3CA* mutation, and hot spot mutation analysis revealed *TP53* mutation, *RB1* mutation, *JUN* amplification. Therapy with temsirolimus and metformin was advised. Patient was alive at last follow up at age 23 years. Immunohistochemical analysis of the patient’s tumor revealed mixed expression of *PTEN*, with approximately two-thirds of the tumor cells having loss and one-third having reduced *PTEN* expression.

Patient 8 was a Caucasian female diagnosed with osteosarcoma of the right distal femur at age 9 years. Patient did not responded to initial regimen with cisplatin, doxorubicin, and high-dose methotrexate and she required an above-the-knee amputation of the right leg. Pathological analysis revealed less than 20% tumor necrosis. Adjuvant chemotherapy consisted of 1 cycle of cisplatin and doxorubicin followed by 6 cycles of ifosfamide and etoposide. The patient had no evidence of disease after this therapy. She also received 24 doses of liposome-encapsulated MTP-PE before she was found to have multiple pulmonary metastases, which were removed via bilateral thoracotomy. She received sunitinib and rapamycin but had problems with thoracotomy wound healing, and her sunitinib had to be discontinued. She was off treatment for approximately 2 months and developed multiple bone metastases. She received gemcitabine, radiation to the bone metastases, and hydroxyurea. She was then found to have a calcified nodule on her stump and underwent local radiotherapy; temozolomide was used as a radiosensitizer. She still had disease progression, mainly in the bones, and was treated with bevacizumab and bortezomib. The patient died of disease progression at age 11 years. Morphoproteomic analysis (Figure 1) of the bone specimen (metastases) revealed expression of *p-ERK1/2* (variable expression [1-3%] of the *RAS/RAF* kinase/extracellular signal–regulated kinase *ERK* pathway, as evidenced by the cytoplasmic expression and nuclear translocation of *p-ERK1/2* in more than 80% of the tumor cells), p-mTOR pathway activation, HSP90 expression, moderate expression of *TRAIL* and *NESTIN* expression.

Patient 9 was a Caucasian female diagnosed with osteosarcoma of the left distal femur at age 18 years. She was initially treated with standard cisplatin, doxorubicin, and high-dose methotrexate. After limb salvage surgery, she was found to have only 25% tumor necrosis. Therefore, she received chemotherapy with ifosfamide and etoposide; after completing chemotherapy, she had no evidence of disease for more than 1 year. The patient was then found to have bilateral lung nodules that were removed via thoracoscopy. She was treated with topotecan and cyclophosphamide but did not tolerate this chemotherapy. She was then found to have osseous, soft tissue, and possible pulmonary metastases. The patient received radiation for local disease control with concomitant temozolomide. After this, she received sorafenib for approximately 1.5 months. Despite chemotherapy, the patient had progressive disease and was referred to our Phase 1 Clinical Trial Service for evaluation. Sequencing of her tumor revealed no pathway aberrations. She was treated on a protocol containing bevacizumab, temsirolimus, and sorafenib. While on this regimen, the patient had progressive disease and died at age 21 years.

Patient 10 was a Caucasian male diagnosed with osteosarcoma affecting the right fibula at 14 years of age. After limb salvage surgery pathology revealed only 30% tumor necrosis; the patient had local recurrence 4 months later, and he went above-the-knee amputation of the right leg. He was then treated with high-dose methotrexate, doxorubicin, cisplatin, ifosfamide, and etoposide. He then received gemcitabine and docetaxel but developed lung metastasis that were resected with eventual development of additional lung metastases. The patient received robatumumab, an anti–insulin-like growth factor 1 receptor antibody, but still had disease progression in the lungs. Had only one dose of LMTP- PE but discontinued this therapy after only 1 dose as new osseous and lung metastases were found. He received radiation to the lungs and started a regimen of high-dose methotrexate, ifosfamide, gemcitabine, rapamycin, and metformin with persistent disease progression in the lungs. Chemotherapy was discontinued, and the patient died at 15 years of age. Morphoproteomic analysis of his primary tumor specimen revealed expression of *p-ERK1/2* (focal and variable expression of the *ERK* pathway as evidenced by the expression and nuclear translocation *of p-ERK1/2*), activation of the *p-mTOR* pathway, expression of *HSP90*, and expression of *TRAIL*, NESTIN, and insulin-like growth factor 1 receptor (*IGFR*).

Patient 11 was a Caucasian male diagnosed with nonmetastatic chondroblastic osteosarcoma of the right distal femur at age 17 years. At his initial presentation, the patient had a large pneumomediastinum. He was treated with methotrexate, cisplatin, and doxorubicin; pathological analysis following limb salvage surgery revealed 80% tumor necrosis. He completed 22 weeks of the methotrexate, cisplatin, and doxorubicin regimen before he voluntarily decided to discontinue treatment. One and a half years after initial diagnosis patient was found to have lung metastasis. Patient had disease progression after 1 cycle of ifosfamide and was treated with doxorubicin and cisplatin. The patient then underwent a left thoracotomy and received intrapleural cisplatin. He then started therapy with metformin and rapamycin, and his disease stabilized. Crizotinib was added to this regimen; however, he had disease progression on this chemotherapy and was referred to our service where tumor sequencing (lung metastases) revealed *MET* amplification, *PI3KCA V344G* mutation, *CCNE1* amplification, and PTPRD S1845fs*2 mutation. The patient was offered the option to enroll in a phase I study of a c-Met inhibitor, but he declined and decided to get therapy elsewhere. Patient died at 19 years of age.

Patient 12 was a Caucasian female diagnosed with osteoblastic osteosarcoma of the right ilium and acetabulum with multiple bone metastases at age 19 years. She was initially treated with cisplatin and doxorubicin, ifosfamide and etoposide and high-dose methotrexate. She received local radiation to the primary tumor and concomitant ifosfamide. She also received 1 dose of samarium 153. The patient received gemcitabine and bevacizumab but had to discontinue this therapy owing to anemia and thrombocytopenia. She then received rapamycin and sunitinib for a short period but discontinued the therapy owing to its side effects. Because she was found to be HER2/neu-positive, she received treatment with *HER2/neu* chimeric antigen receptor T cells. Despite this therapy, she had disease progression with worsening bone metastases, and she was started on denosumab. At this point she was referred to our service and a met- inhibitor was offered but patient could not start it due to thrombocytopenia. She was treated with lapatinib and metformin but she had disease progression that included skull metastases, and her regimen was switched to one consisting of doxorubicin and denosumab. The patient required radiotherapy to treat skull metastases and had concomitant temozolomide during radiotherapy. After this she had osseous progression in spine and skull and patient received samarium 153, and then had infusion of autologous stem cells. Few months after that patient underwent removal of right frontal skull and dural metastases. At this time, molecular profiling of the patient primary tumor was performed and it revealed *FGFR1* overexpression, SOX2 amplification and no PDGFRA amplification. Profiling with immunohistochemistry and PCR-based panels revealed the tumor to be positive for *TL3*, a member of the transducing-like enhancer of split (*TLE)*, negative for monoclonal and polyclonal SPARC. Patient started on a regimen containing protein-bound paclitaxel (to suppress *TLE3*) and gemcitabine but patient progressed on this regimen and was switched to liposomal doxorubicin. She received radiation to the orbit and clavicle with concomitant pazopanib and had good response to this. She was then started on chemotherapy with monthly liposomal doxorubicin and patient decided to continue treatment elsewhere. Patient was 24 years old on last clinic visit.

Patient 13 was a 19-year-old Caucasian female with osteosarcoma of the left distal femur. At diagnosis, she was found to have activity on bone scan in the right iliac wing, T9, and left mandible. T9 and her right iliac wing were biopsied and were both positive for osteosarcoma. She was referred to phase 1 clinic after several lines of standard chemotherapy, limb salvage surgery, radiation treatment, left thoracotomy, and most radiation treatment to T9 and the left sacroiliac region. Her tumor was positive for CD30 by immunohistochemistry. She could not be enrolled on a clinical trial with an anti-CD 30 anti-body drug conjugate as her platelets were low and performance status was declining. She was given Brentuximab as a palliative therapy.

## DISCUSSION

We have reported one of the first clinical series of theranostic profiling of advanced osteosarcoma to explore actionable aberrations in heavily pre-treated patients. The most common actionable aberrations in our cohort occurred in the *PI3K/Akt/mTOR* pathway. No patterns in genomic alterations beyond the above were readily identifiable, and suggest both high molecular diversity in osteosarcoma and the need for more analysis to define distinct subgroups of osteosarcoma defined by genomic alterations. The recognition of heterogeneity of the biology of this disease gained from this study provides critical information as it applies to evolving research practices using molecular profiling and/or patient care in osteosarcoma. Based on these observations we hypothesize that the biology of aggressive osteosarcoma is akin to fingerprints, in that no two tumors are alike. This hypothesis needs to be tested in larger datasets of osteosarcoma patients using matching primary and sequential metastastic samples over time.

In addition to the PI3K/Akt/mTOR pathway aberrations, we have have also demonstrated several other diverse aberrations like *TP53* truncation and mutation*, PTPRD*, *MET* amplification, *BCL2L2* amplification, and *NKX2-1* amplification. In addition we report CD 30 positivity in osteosarcoma which is most commonly seen in Hodgkin’s lymphoma.

In the present study *PTEN* loss was the most common molecular defect detected in tumor samples. *PTEN* encodes a phosphatase that functions as a tumor suppressor by negatively regulating the *PI3K/Akt/mTOR* pathway. Loss of PTEN by mutation or deletion has been correlated with decreased survival in patients with several different tumor types. In breast cancer PTEN activation contributes to trastuzumab’s antitumor activity. Patients with PTEN-deficient breast cancers had significantly poorer responses to trastuzumab-based therapy than those with normal PTEN. Additionally, PI3K inhibitors rescued PTEN loss-induced trastuzumab resistance, suggesting that PI3K-targeting therapies could overcome this resistance[11].

Biallelic and monoallelic deletion of PTEN has been observed in 15% and 33% of osteosarcomas, respectively [12]. The loss or inactivation of PTEN leads to the activation of PI3K and its downstream signaling pathway (the PI3KCA/Akt/mTOR pathway) and may predict sensitivity to inhibitors of mTOR (e.g., temsirolimus, everolimus) or PI3K itself. We also found PIK3CA mutations. PI3K is a lipid kinase that is a known regulator of cellular growth and proliferation whose pathway plays a critical role in cancer development [13]. Choy et al. were the first to identify several mutations involving the *PI3K* pathway in a large mutational profile of osteosarcoma tumor samples [14]. Activating *PIK3CA* mutations may predict sensitivity to *PI3K/Akt/mTOR* pathway inhibitors [15]. Therefore, the role of *PIK3/AKT/mTOR* pathway regulators needs to be explored in osteosarcoma patients.

*RB1* encodes the retinoblastoma protein, a tumor suppressor and negative cell cycle regulator. *RB1* loss or mutation has been reported in a variety of solid tumors, including osteosarcoma. The *RB1* tumor suppressor gene plays a key role in osteoblast and bone formation, and deletion or inactivation of *RB1* is a frequent occurrence in osteosarcoma. In knockout mice, loss of pRb increase the osteoprogenitor cells and reduced terminal differentiation and cell cycle exit. This increased pool of osteoprogenitor cells may be susceptible to additional transforming events leading to osteosarcoma [16]. No therapies targeting *RB1* loss are currently available.

We detected *p53* mutation in 1 patient and *p53* truncation in 1 patient. *p53* is a tumor suppressor that is encoded by the *TP53* gene, and its functional loss is common in aggressive advanced cancers[17]. Germline carriers of *TP53* mutations have a higher incidence of sarcoma, including osteosarcoma[18]. p53 truncation leads to a loss of function via the loss of the transactivation of *p53*-dependent genes and the loss of the tetramerization domain. No approved therapies targeting *TP53* mutations are available. p53 mutations have been reported in osteosarcomas, but to our knowledge, we are the first to report such mutations in osteoblastic tumors[17, 18].

*MYC* amplification in osteosarcoma is common, and MYC expression in this tumor has been correlated with poor prognosis [19]. Also contributes to tumorigenesis by inducing genomic instability. [20] Although no currently available therapies can directly target *MYC,* preclinical evidence suggests that the sorafenib derivative SC-1 downregulates *c-Myc* in osteosarcoma cells, and this agent could be a future treatment option for patients with tumors that have *MYC* amplification. SC-1’s mechanism of action involves the downregulation of the signal transducer and activator of transcription 3 (STAT3) and the expression of its driver genes, including *cyclin-D* and *c-Myc* [21].

*MET* encodes a receptor tyrosine kinase whose activation by the ligand hepatocyte growth factor results in signaling via the *RAS/RAF/MAPK* and *PI3K* pathways to promote tumor proliferation and survival. *MET* amplification has been associated with poor prognosis in patients with gastric, lung, and pancreatic cancers [22- 24]. To our knowledge, *MET* amplification has not been previously reported in osteosarcoma. Evidence shows that overexpression of the MET oncogene in osteoblasts resulted in the conversion of primary human osteoblasts into osteosarcoma cells, displaying the transformed phenotype in vitro and the distinguishing features of human osteosarcomas in vivo. [25] *MET* amplification may predict sensitivity to *MET* inhibitors such as crizotinib, a dual *Alk/Met* inhibitor that is approved by the Food and Drug Administration for the treatment of patients with *ALK*-positive non–small cell lung cancer. Crizotinib has also elicited clinical responses in patients with *MET*-amplified gastroesophageal cancer and *MET*- amplified non–small cell lung cancer [26, 27].

*CCNE1* encodes the cyclin E1 protein, which activates cyclin-dependent protein kinase 2 and plays a role in regulating cells’ transition from G1 to S phase. Cyclin E1 also has a direct role in initiating the replication and maintenance of genomic stability. Lockwood et al. previously reported *CCNE1* amplification, which is highly associated with cyclin E1 expression and can lead to cell transformation in osteosarcoma, but did not find it to be correlated with therapy response or clinical outcome [28]. No approved therapies targeting cyclin E1 are available.

*PTPRD* encodes a transmembrane protein tyrosine phosphatase that acts as a tumor suppressor by dephosphorylating the oncoprotein *STAT3*. *PTPRD* mutations have not been reported previously in osteosarcoma or other bone tumors. In other tumors, however, *PTPRD* alterations may be linked with cancer development and metastases, and reduced expression of *PTPRD* has been reported to be associated with poor prognosis [29-31]. There are currently no approved treatments targeting *PTPRD* mutations. Given that *PTPRD* inactivation may increase *STAT3* activity, the use of *STAT3* inhibitors may have relevance in the treatment of osteosarcomas with *PTPRD* mutations[32].

*BCL2L2* encodes Bcl-w, a pro-survival protein that mediates apoptosis. *BCL2L2* amplification in osteosarcoma has not been reported previously, but 25% of osteosarcomas express the related protein Bcl-2, which has been linked with decreased long-term survival [19]. *BCL2L2* amplification in non–small cell lung cancer has been associated with poor prognosis [33]. No current approved therapies target amplification or overexpression of *BCL2L2*.

The *NKX2-1* gene encodes thyroid transcription factor 1 (TTF-1). NKX2-1 amplification causes the overexpression of TTF-1 and upregulates the transcription of downstream target genes [34]. To our knowledge, NKX2-1 amplification in osteosarcoma has not been reported previously. TTF-1 is expressed in the majority of lung adenocarcinomas (where its overexpression is associated with poor prognosis) as well as in small cell carcinomas and a subset of thyroid and central nervous system tumors and also in T cell acute lymphoblastic leukemia [35-37] No approved therapies targeting TTF-1 expression are available.

Table 3 shows molecular aberration found in our patients and possible therapies to target these aberrations (FDA approved or used in clinical trials).

**Table 3:** Molecular aberrations identified in 12 osteosarcoma patients with potential targeted therapies

Aberration	Previous reports	No. reported in present study	Mutation	Pathway	FDA-approved therapies	Potential therapies in clinical trials
*PTEN* loss	Yes	3		*PI3KCA/mTOR* pathway	Sirolimus, everolimus, temsirolimus	PIK3CA inhibitors, AKT inhibitors, mTOR inhibitors
*P53* trun	Yes (No for osteoblastic tumors)	1	N131fs*39	Apoptosis pathway	Bevacizumab	
*P53* mut	Yes	1				
*RB1* loss	Yes	1	S308fs*6		None	Aurora Kinase inhibitors, BCL- 2 inhibitors, Notch Pathway inhibitors
*RB1* mut		1				
*PIK3CA* mut	Yes	2	*M1043VV344G*	*PI3KCA/mTOR* pathway	Sirolimus, everolimus, temsirolimus	PIK3CA inhibitors, AKT inhibitors, mTOR inhibitors
*PTPRD* mut	No	1	S1845fs*2		None	None
*MET* amp	No	1		*Ras/Raf/Mapk* and *PI3K/mTOR* pathways	Crizotinib	MET inhibitors
*EGFR* amp		1			Erlotinib, gefitinib, cetuximab panitumumab, lapatinib	EGFR receptor blockers, EGFR tyrosine kinase inhibitors
*MYC* amp	Yes	1			None	CDK inhibitors, Aurora Kinase inhibitors
*JUN* amp	Yes	1			None	MMP inhibitors
*BCL2L2* amp	No	1		Apoptosis pathway	None	BCL –w inhibitors
*NKX2-1* amp	No	1			None	None
*CCNE1* amp	Yes	1		Cell cycle	None	None

The limitations of this study are its retrospective nature, profiling of the aggressive phenotype at different time points, and heterogeneity of approach to identify aberrations. However, identification of actionable aberrations in a CLIA compliant lab could benefit patients if an actionable aberration is found. Most of the previous studies have used an approach that consists of sequential screening for individual aberrations. But given the diversity of an orphan disease like osteosarcoma, such a comprehensive theranostic technology agnostic approach is likely to be more efficient and informative than a single gene test or a specific assay. In addition patients with high risk, aggressive biology disease may need to be identified much earlier in their disease course when a targeted therapy option could be more effective than when a disease is multiply relapsed.

## PATIENTS & METHODS

We retrospectively reviewed the medical records of patients with advanced metastatic osteosarcoma who were referred to the Department of Investigational Cancer Therapeutics (Phase I Clinical Trials Program) and/ or Division of Pediatrics between 6/1/2008 and 02/01/2013 and for whom archival tumor tissue samples were available. Tissue samples could be either from primary tumor or from metastatic focus, mainly from the lungs.

The University of Texas MD Anderson Cancer Center Institutional Review Board approval was obtained prior to performing this study, and all patients provided written informed consent for participation and for the chemotherapy or targeted therapy they received.

Eligible patients were those who had been referred to our Phase 1 Clinical Trial Service to receive available targeted therapies or to enroll in clinical trials of targeted therapies against osteosarcoma. Patients’ prior treatments were recorded upon their initial evaluation at our center. Demographic data including age, gender, and ethnicity were collected. Other information such as age at initial presentation, location of primary tumor, histology, sites of metastases was also recorded.

### Specimen analysis

Several methods were used to analyze the patients’ tumor archival samples and varied according to the CLIA testing available at the time of referral. Next-generation sequencing–based (NGS) assays were performed by Foundation Medicine (Boston, MA) to identify genomic alterations within 186 cancer-related genes. Immunohistochemistry- and polymerase chain reaction (PCR)-based panels were performed by CARIS Target Now (CARIS Life Sciences, Irving, TX, and Phoenix, AZ) or in a CLIA-compliant laboratory at MD Anderson. Morphoproteomic analysis, which incorporate morphology and proteomics to identify therapy customized to individual patients [7], were performed by Dr. Robert Brown in the Department of Pathology and Laboratory Medicine at The University of Texas Health Science Center at Houston. Comparative genome hybridization was performed by Oncopath Laboratory in San Antonio, TX. In addition hotspot genomic aberrations using Clinical Laboratory Improvement Amendments (CLIA)-certified Molecular Diagnostics Laboratory at MD Anderson using standard operating procedures and PCR-based sequencing technology for all tests was done as a screening test whenever a protocol warranted the test as a screening test for a specific clinical trial. One patient had CD30 immunohistochemistry based test sent to Quest diagnostics as a part of screening for a specific trial.

### Statistical analysis

This is a descriptive study with no formal hypothesis testing. We used descriptive statistics to summarize our findings.

## CONCLUSIONS

In this hypothesis generating study, based on our preliminary observations we hypothesize that aggressive and the metastatic phenotype osteosarcoma at the genomic and proteomic level is similar to human fingerprints, in that no two tumors are indistinguishable. In addition, our findings underscore the importance of performing tumor profiling earlier in the disease course in osteosarcoma patients. Doing so provides valuable information about the aberrant pathways triggering tumor growth and metastasis. Although tumor profiling does not always reveal a targetable genomic aberration, molecular characterization of the tumor may provide valuable insight into its behavior. Ideally, noninvasive methods such as plasma proteomic profiling would be used to profile tumors in osteosarcoma patients at diagnosis to identify prognostic biomarkers and targetable genomic aberrations. Further large-scale studies are needed to investigate the clinical benefit of using commercially available profiling services to match patients who have targetable aberrations with the appropriate therapies and also to prove our hypothesis of fingerprint-like genomic behavior of metastatic osteosarcoma.

In summary, osteosarcoma presents with high molecular diversity emphasizing the need for more analyses to define distinct subgroups of osteosarcoma defined by genomic alterations. Early theranostic profiling in high risk osteosarcoma patients may identify prognostic biomarkers and targetable genomic alterations. Further large-scale investigations are needed to evaluate the clinical benefit of matching patients with actionable aberrations.

## References

[1] Pappo AS (2006). Pediatric bone and soft tissue sarcomas.

[2] Cleton-Jansen AM, Anninga JK, Briaire-de Bruijn IH, Romeo S, Oosting J, Egeler RM, Gelderblom H, Taminiau AH, Hogendoorn PC (2009). Profiling of high-grade central osteosarcoma and its putative progenitor cells identifies tumourigenic pathways. British journal of cancer.

[3] Anderson PM, Pearson M (2006). Novel therapeutic approaches in pediatric and young adult sarcomas. Curr Oncol Rep.

[4] Anderson P, Kopp L, Anderson N, Cornelius K, Herzog C, Hughes D, Huh W (2008). Novel bone cancer drugs: investigational agents and control paradigms for primary bone sarcomas (Ewing’s sarcoma and osteosarcoma). Expert Opin Investig Drugs.

[5] van Oosterwijk JG, Anninga JK, Gelderblom H, Cleton- Jansen AM, Bovee JV (2013). Update on Targets and Novel Treatment Options for High-Grade Osteosarcoma and Chondrosarcoma. Hematol Oncol Clin North Am.

[6] Hong DS, Said R, Falchook GS, Naing A, Moulder SL, Tsimberidou AM, Galluppi G, Dakappagari N, Storgard C, Kurzrock R, Rosen LS (2013). Phase I Study of BIIB028, a selective heat shock protein 90 inhibitor, in patients with refractory metastatic or locally advanced solid tumors. Clin Cancer Res.

[7] Brown RE (2009). Morphogenomics and morphoproteomics: a role for anatomic pathology in personalized medicine. Archives of pathology & laboratory medicine.

[8] Tsimberidou AM, Iskander NG, Hong DS, Wheler JJ, Falchook GS, Fu S, Piha-Paul S, Naing A, Janku F, Luthra R, Ye Y, Wen S, Berry D, Kurzrock R (2012). Personalized medicine in a phase I clinical trials program: the MD Anderson Cancer Center initiative. Clin Cancer Res.

[9] Flaherty KT, Puzanov I, Kim KB, Ribas A, McArthur GA, Sosman JA, O'Dwyer PJ, Lee RJ, Grippo JF, Nolop K, Chapman PB (2010). Inhibition of mutated, activated BRAF in metastatic melanoma. N Engl J Med.

[10] Kwak EL, Bang YJ, Camidge DR, Shaw AT, Solomon B, Maki RG, Ou SH, Dezube BJ, Janne PA, Costa DB, Varella-Garcia M, Kim WH, Lynch TJ, Fidias P, Stubbs H, Engelman JA (2010). Anaplastic lymphoma kinase inhibition in non-small-cell lung cancer. N Engl J Med.

[11] Nagata Y, Lan KH, Zhou X, Tan M, Esteva FJ, Sahin AA, Klos KS, Li P, Monia BP, Nguyen NT, Hortobagyi GN, Hung MC, Yu D (2004). PTEN activation contributes to tumor inhibition by trastuzumab, and loss of PTEN predicts trastuzumab resistance in patients. Cancer cell.

[12] Freeman SS, Allen SW, Ganti R, Wu J, Ma J, Su X, Neale G, Dome JS, Daw NC, Khoury JD (2008). Copy number gains in EGFR and copy number losses in PTEN are common events in osteosarcoma tumors. Cancer.

[13] Muller CI, Miller CW, Hofmann WK, Gross ME, Walsh CS, Kawamata N, Luong QT, Koeffler HP (2007). Rare mutations of the PIK3CA gene in malignancies of the hematopoietic system as well as endometrium, ovary, prostate and osteosarcomas, and discovery of a PIK3CA pseudogene. Leukemia research.

[14] Choy E, Hornicek F, MacConaill L, Harmon D, Tariq Z, Garraway L, Duan Z (2012). High-throughput genotyping in osteosarcoma identifies multiple mutations in phosphoinositide-3-kinase and other oncogenes. Cancer.

[15] Janku F, Tsimberidou AM, Garrido-Laguna I, Wang X, Luthra R, Hong DS, Naing A, Falchook GS, Moroney JW, Piha-Paul SA, Wheler JJ, Moulder SL, Fu S, Kurzrock R (2011). PIK3CA mutations in patients with advanced cancers treated with PI3K/AKT/mTOR axis inhibitors. Molecular cancer therapeutics.

[16] Gutierrez GM, Kong E, Sabbagh Y, Brown NE, Lee JS, Demay MB, Thomas DM, Hinds PW (2008). Impaired bone development and increased mesenchymal progenitor cells in calvaria of RB1−/− mice. Proceedings of the National Academy of Sciences of the United States of America.

[17] Fu HL, Shao L, Wang Q, Jia T, Li M, Yang DP (2013). A systematic review of p53 as a biomarker of survival in patients with osteosarcoma. Tumour Biol.

[18] Osumi T, Miharu M, Fuchimoto Y, Morioka H, Kosaki K, Shimada H (2012). The germline TP53 mutation c.722 C>T promotes bone and liver tumorigenesis at a young age. Pediatr Blood Cancer.

[19] Wu X, Cai ZD, Lou LM, Zhu YB (2012). Expressions of p53, c-MYC, BCL-2 and apoptotic index in human osteosarcoma and their correlations with prognosis of patients. Cancer epidemiology.

[20] Felsher DW, Bishop JM (1999). Transient excess of MYC activity can elicit genomic instability and tumorigenesis. Proceedings of the National Academy of Sciences of the United States of America.

[21] Wang CT, Lin CS, Shiau CW, Chu PY, Hsiao CC, Chiang YL, Tai WT, Chen KF (2013). SC-1, a sorafenib derivative, shows anti-tumor effects in osteogenic sarcoma cells. Journal of orthopaedic research : official publication of the Orthopaedic Research Society.

[22] Graziano F, Galluccio N, Lorenzini P, Ruzzo A, Canestrari E, D'Emidio S, Catalano V, Sisti V, Ligorio C, Andreoni F, Rulli E, Di Oto E, Fiorentini G, Zingaretti C, De Nictolis M, Cappuzzo F (2011). Genetic activation of the MET pathway and prognosis of patients with high-risk, radically resected gastric cancer. Journal of clinical oncology : official journal of the American Society of Clinical Oncology.

[23] Park S, Choi YL, Sung CO, An J, Seo J, Ahn MJ, Ahn JS, Park K, Shin YK, Erkin OC, Song K, Kim J, Shim YM, Han J (2012). High MET copy number and MET overexpression: poor outcome in non-small cell lung cancer patients. Histology and histopathology.

[24] Zhu GH, Huang C, Qiu ZJ, Liu J, Zhang ZH, Zhao N, Feng ZZ, Lv XH (2011). Expression and prognostic significance of CD151, c-Met, and integrin alpha3/alpha6 in pancreatic ductal adenocarcinoma. Digestive diseases and sciences.

[25] Patane S, Avnet S, Coltella N, Costa B, Sponza S, Olivero M, Vigna E, Naldini L, Baldini N, Ferracini R, Corso S, Giordano S, Comoglio PM, Di Renzo MF (2006). MET overexpression turns human primary osteoblasts into osteosarcomas. Cancer research.

[26] Lennerz JK, Kwak EL, Ackerman A, Michael M, Fox SB, Bergethon K, Lauwers GY, Christensen JG, Wilner KD, Haber DA, Salgia R, Bang YJ, Clark JW, Solomon BJ, Iafrate AJ (2011). MET amplification identifies a small and aggressive subgroup of esophagogastric adenocarcinoma with evidence of responsiveness to crizotinib. Journal of clinical oncology : official journal of the American Society of Clinical Oncology.

[27] Ou SH, Kwak EL, Siwak-Tapp C, Dy J, Bergethon K, Clark JW, Camidge DR, Solomon BJ, Maki RG, Bang YJ, Kim DW, Christensen J, Tan W, Wilner KD, Salgia R, Iafrate AJ (2011). Activity of crizotinib (PF023410), a dual mesenchymal-epithelial transition (MET) and anaplastic lymphoma kinase (ALK) inhibitor, in a non-small cell lung cancer patient with de novo MET amplification. Journal of thoracic oncology : official publication of the International Association for the Study of Lung Cancer.

[28] Lockwood WW, Stack D, Morris T, Grehan D, O’Keane C, Stewart GL, Cumiskey J, Lam WL, Squire JA, Thomas DM, O'Sullivan MJ (2011). Cyclin E1 is amplified and overexpressed in osteosarcoma. The Journal of molecular diagnostics : JMD.

[29] Kohno T, Otsuka A, Girard L, Sato M, Iwakawa R, Ogiwara H, Sanchez-Cespedes M, Minna JD, Yokota J (2010). A catalog of genes homozygously deleted in human lung cancer and the candidacy of PTPRD as a tumor suppressor gene. Genes, chromosomes & cancer.

[30] Funato K, Yamazumi Y, Oda T, Akiyama T (2011). Tyrosine phosphatase PTPRD suppresses colon cancer cell migration in coordination with CD44. Experimental and therapeutic medicine.

[31] Veeriah S, Brennan C, Meng S, Singh B, Fagin JA, Solit DB, Paty PB, Rohle D, Vivanco I, Chmielecki J, Pao W, Ladanyi M, Gerald WL, Liau L, Cloughesy TC, Mischel PS (2009). The tyrosine phosphatase PTPRD is a tumor suppressor that is frequently inactivated and mutated in glioblastoma and other human cancers. Proceedings of the National Academy of Sciences of the United States of America.

[32] Jiang Y, Janku F, Subbiah V, Angelo LS, Naing A, Anderson PM, Herzog CE, Fu S, Benjamin RS, Kurzrock R (2013). Germline PTPRD mutations in Ewing sarcoma: biologic and clinical implications. Oncotarget.

[33] Kawasaki T, Yokoi S, Tsuda H, Izumi H, Kozaki K, Aida S, Ozeki Y, Yoshizawa Y, Imoto I, Inazawa J (2007). BCL2L2 is a probable target for novel 14q11.2 amplification detected in a non-small cell lung cancer cell line. Cancer science.

[34] Kwei KA, Kim YH, Girard L, Kao J, Pacyna-Gengelbach M, Salari K, Lee J, Choi YL, Sato M, Wang P, Hernandez- Boussard T, Gazdar AF, Petersen I, Minna JD, Pollack JR (2008). Genomic profiling identifies TITF1 as a lineage-specific oncogene amplified in lung cancer. Oncogene.

[35] Moldvay J, Jackel M, Bogos K, Soltesz I, Agocs L, Kovacs G, Schaff Z (2004). The role of TTF-1 in differentiating primary and metastatic lung adenocarcinomas. Pathology oncology research : POR.

[36] Gilbert-Sirieix M, Massaad-Massade L (2011). [TTF-1: neither angel nor demon]. Medecine sciences : M/S.

[37] Homminga I, Pieters R, Langerak AW, de Rooi JJ, Stubbs A, Verstegen M, Vuerhard M, Buijs-Gladdines J, Kooi C, Klous P, van Vlierberghe P, Ferrando AA, Cayuela JM, Verhaaf B, Beverloo HB, Horstmann M (2011). Integrated transcript and genome analyses reveal NKX2-1 and MEF2C as potential oncogenes in T cell acute lymphoblastic leukemia. Cancer cell.

